# Adolescent selfie: an Italian Society of Paediatrics survey of the lifestyle of teenagers

**DOI:** 10.1186/s13052-019-0653-7

**Published:** 2019-05-17

**Authors:** Martina Smorti, Annarita Milone, José Gonzalez Gonzalez, Giovanni Vitali Rosati

**Affiliations:** 1 Department of Surgical, Medical and Molecular Pathology and Critical Care, University of Pisa, Via Savi 10, 56126 Pisa, Italy; 2 IRCCS Stella Maris, Scientific Institute of Child Neurology and Psychiatry, Calambrone, Pisa, Italy; 3 Paediatric Department, USL Toscana Centro, Florence, Italy; 4 USL Toscana Centro, Florence, Italy

**Keywords:** Adolescents, Body perception, Sexual behaviour, Mental health, Internalizing problems, Externalizing problems, Family violence

## Abstract

**Background:**

Epidemiological studies worldwide indicate that teenagers are at risk of internalizing and externalizing problems that persist into adulthood. In our country, there are few epidemiological studies on adolescents internalizing and externalizing problems. These studies, however, were not conducted in all of Italy. The aim of this study, promoted by the Italian Society of Paediatrician (SIP), was to investigate: a) the lifestyle and the prevalence of internalizing and externalizing problems in Italian teenagers and b) the risk and protective factors in family and social contexts. A further aim was to analyse gender differences in the above-mentioned variables.

**Methods:**

11,527 adolescents aged 13 to 21 years were recruited among students of Italian high schools. Participants were contacted by school authorities inviting them to participate in an internet survey on youth health and lifestyle. If they agreed to participate, the adolescents filled out an on-line anonymous questionnaire. The questionnaire was composed of 60 multiple choice items to investigate nutrition, body perception and lifestyle, internet exposure and sexual behaviour, externalizing and internalizing problems, family context and social context. Participation in the study was completely voluntary. The statistical significance of gender differences was tested by means of Chi-square analyses. Results displayed that Italian female adolescents are at risk of internalizing problems while males are at higher risk of externalizing problems. Moreover, several risk factors emerged from the family context in terms of violence, physical and psychological abuse.

**Conclusions:**

It is critical to make paediatricians and schools aware of the main points to improve prevention and healthcare in the teenage population. To our knowledge, this is the first study in the Italian context to enrol more than 11,000 adolescents.

## Background

Epidemiological studies worldwide indicate that up to one in five children and teenagers experience mental health problems that persist into adulthood [1, 2]. Up to one half of all mental health problems have their onset before the age of 14, and mental illnesses that emerge before adulthood impose a 10-fold higher cost than those that emerge later in life [3].

The presence of mental health problems in children and teenagers negatively influences their well-being, causing internalizing and externalizing problems. In psychology, the distinction between internalizing and externalizing problems is often used to distinguish symptoms directed to oneself (or internalizing), such as depression, anxiety and social withdrawal, from behaviours outer-directed (or externalizing), such as rule-breaking, aggression, impulsivity, and defiance [4, 5]_._

The relevance of externalizing problems may be explained with the fact that adolescence is physiologically characterized by an amplification of desire, the impulse for new experiences, and the research of extreme emotions (sensation seeking, novelty seeking, exploration). The complex set of neurobiological, neuropsychological, emotional and relational characteristics typical of adolescence can favour access to new experiences, which may include an initiation to alcohol and the use of other drugs [6].

Internalizing and externalizing problems, which are associated with lower academic achievement, school failure, social rejection and marginality [7, 8], affect adolescent males and females differently. In fact, while internalizing problems seem to be especially prevalent among girls, externalizing disorders seem to be common among boys [1, 9]_._

Several risk factors have been identified for the health of children and teenagers. It has been shown that poor economic conditions (lower socio-economic status, worsening of economic inequality), a poor family context (single parenting, parental conflicts, parental mental health problems, parental disorders due to abuse of substances), a negative parenting style (lack of monitoring and involvement, harsh parenting, neglect, victimization), as well as the affiliation to deviant peer context, constitute significant risk factors for the development of internalizing and externalizing problems [10, 11]_._

Despite the widespread recognition of the importance of mental health promotion and prevention in children and teenagers, there is, in many countries, a gap between needs and resource availability. Accumulating evidence suggests that early intervention can provide long-term health and socioeconomic benefits by prevention of the onset of mental health problems and their development into chronic disorders [12].

Although there is an ever-greater definition in nosographic, etiopathogenetic, and neurobiological terms of the onset of clinical entities in the developmental age, there is currently a large series of children that do not come to neuropsychiatric or psychological attention. Sometimes they stop at the first contact with professionals who do not specifically deal with mental health in the developmental age (paediatrician, general practitioner, paramedical, etc.), not accessing the possibility of a diagnostic and therapeutic pathway that could reduce the risk of chronic illnesses and unfavourable prognosis [12, 13]. The data described show us that it is extremely important to disseminate knowledge of the mental health needs of children and teenagers, and the prevalence, semiology, and risk factors of mental health problems. In particular, training and supervision of non-mental health professionals working with children in the identification and management of mental health problems is indicated as a strategy to improve prevention in child and adolescent mental health [13, 14]_._

In our country, there are few epidemiological studies that have attempted to provide a general overall view on mental health in the developmental years and, in particular, regarding the pre-adolescent and adolescent age with a view to assessing areas of particular criticality in the population, with objectives of assessment, prevention and intervention.

In Italy, the PRISMA study [15] estimated a prevalence and correlation of mental health problems in 3418 urban preadolescents, living in areas of central and northern Italy. The study revealed a prevalence of 9,8% of emotional disorders according to DSM-IV and a prevalence of 8,2% of externalizing disorders. The overall prevalence of mental disorders in Italian teenagers was similar to that found in other studies conducted in European countries. However, few studies analysed the prevalence and impact of internalizing and externalizing problems in a sample representative of the whole Italian adolescent population. Moving from previous considerations, the aim of this study was to investigate, in a representative sample of the entire Italian youth population, the lifestyle and the prevalence of internalizing and externalizing problems, and the factors in family and social context that may increase the risk of developing these problems.

## Method

This study was promoted by the Italian Society of Paediatrics and extended from a regional study aimed at investigating the adolescent lifestyle in Tuscany [16]. With the purpose of extending the investigation to the lifestyle of the Italian adolescent population, in order to improve prevention and healthcare in the youth population, we aimed to investigate: a) lifestyle (related to nutrition, internet use, sexual activities and health behaviour) and the prevalence of internalizing and externalizing problems in Italian teenagers, and b) the risk and protective factors in family and social contexts. A further aim of this study was to analyse gender differences in the above-mentioned variables.

### Setting

The study took place from January to June 2017 in school settings. Participants were recruited among high school students (aged 13 or more) belonging to different geographical areas of Italy.

### Materials and procedures

A multidisciplinary working group coordinated by the Italian Society of Paediatrics and made up of paediatricians, psychologists, epidemiologists, child psychiatrists, and sociologists who worked at universities or public health departments, developed an online survey composed of 60 multiple choice questions. The questionnaire was focused on increasing the knowledge of attitudes and practices of a population of teenagers who attended Italian high schools or secondary schools about:

1. Body perception, nutrition and health behaviour: 8 questions to investigate dietary habits (related to breakfast, snack and lunch), the perception of body weight (normal weight, underweight, overweight) reported from the teenagers and from paediatricians, and the practice of sports activities.
2. Internet exposure and sexual behaviour: 10 questions to investigate the age of receiving the first smartphone and the frequency of internet use and misuse (remaining online longer than desired, negative internet influence on studies, perception of finding reliable information online) and the frequency of sexual behaviour (using contraceptives, practicing sex to gain an economic advantage, receiving sexual proposals from adults via dating apps.) (response on a Likert scale from 1 = never to 5 = always).
3. Externalizing and internalizing problems: 10 questions to investigate the frequency of externalizing (to undergo or carry out acts of bullying or cyberbullying, smoking cigarettes, using drugs, abusing alcohol) and internalizing (feelings of intense psychological sufferance, perform acts of self-harm) problems (response on a Likert scale from 1 = never to 5 = always).
4. Family context: 10 questions to investigate: a) the family context of the teenager (with indication of the family members they live with) or non-family (shelter house or refuge for young) (3 items); b) how often they were witnesses of arguments, abuse, or violence in family (3 items, responses on the Likert scale from 1 = never to 5 = always); c) how often they turn to parents in case of problems or difficulties (2 items, response on a Likert scale from 1 = never to 5 = always); d) perception of the quality of relationships with both parents, and attention received in the family (2 items, response on a Likert scale from 1 = very bad to 5 = very good).
5. Social context: 11 questions to investigate how often adolescents: a) believe that social and school contexts are receptive to the youth’s needs, b) perceive support from friends in case of difficulty, c) prefer “on-line” interactions compared to real ones, d) use internet to communicate (response on a Likert scale from 1 = never to 5 = always). In addition, there were two questions to investigate the need for psychological support and the consultation of a psychologist. Finally, one question asked about membership in groups or associations.

In addition to this data, socio-demographic information was collected (gender, age, place of residence, place of birth (Italy / foreign country).

The Italian Society of Paediatricians, involving the regional authorities, contacted all the secondary schools in Italy, inviting them to inform their students about the on-line questionnaire on youth health and lifestyle. The questionnaire was anonymous, and participation was voluntary.

School authorities explained the aim of the study to students and invited them to participate. If they agreed, the adolescents completed an on-line questionnaire.

### Sample

11,527 adolescents (49.7% males) aged from 13 to 21 years (mean age 16.16 s.d. 1.64) were recruited for this study among students of Italian high schools. The breakdown of the sample by geographical area of residence was: 31.6% (3637) central-northern Italy, 30.5% southern Italy (*n* = 3519) and 37.9% the islands area (*n* = 4371). 95% of the participants were born in Italy (10938), while the remaining (*n* = 589) were born in other countries.

### Data analysis

The SPSS 20 for Windows statistical program was used for all analyses.

Because a majority of the questions in sections 2, 3, 4 and 5 were designed to register the frequency by means of a 5-point Likert scale from 1 = never to 5 = always, we decided to recode the answers of the questionnaire in three alternatives 1 = never; 2 = occasionally (answer 2 = rarely and 3 = sometimes); 3 = usually (answer 4 = very often and 5 = always). Moreover, because the answers concerning the perception of the quality of the relationship with parents were designed on a Likert scale from 1 = very bad to 5 = very good, we decided to recode the answers of the questionnaire in three dimensions 1 = negative (answer 1 = very bad and 2 = bad); 2 = neutral; 3 = positive (answer4 = good and 5 = very good).

The statistical significance of gender differences was tested by means of Chi-square analyses. Statistical significance was considered for *p* value <.05.

## Results

The results will be presented in thematic areas:

1. Body perception, nutrition and health behaviours

Table 1 displays the gender distribution of body perception, nutrition and health behaviour variables in the study population.

**Table 1:** Frequency and percentage for body perception, nutrition and health behaviours variables according to gender

		Males n %	Females n %	Total n %
Your pediatrician thinks your weigh is	Underweigh	414	479	893
		46,40%	53,60%	*7,70%*
	Regular	4650	4657	9307
		50,00%	50,00%	*80,70%*
	Overweigh	660	667	1327
		49,70%	50,30%	*11,50%*
What do you think about your weigh?	Underweigh	556	260	816
		68,10%	31,90%	*7,10%*
	Regular	4159	3361	7520
		55,30%	44,70%	*65,20%*
	Overweigh	1009	2182	3191
		31,60%	68,40%	*27,70%*
Do you have breakfast at home?	Never	1139	1469	2608
		47,30%	56,30%	*22,60%*
	Sometimes	1222	1475	2697
		45,30%	54,70%	*23,40%*
	Always	3363	2859	6222
		54,10%	45,90%	*54,00%*
Do you have a snack when you are at school?	Never	884	1010	1894
		46,70%	53,30%	*16,40%*
	almost time	4840	4793	9633
		50,25%	49,75%	*83,60%*
Do you attend sport activities at the moment?	No	2162	3212	5374
		40,20%	59,80%	*46,60%*
	Yes	3562	2591	6153
		57,90%	42,10%	*53,40%*

Note. Percentage are reported for males and females while percentage in *italic* are reported for total sample

According to paediatricians, the majority (80,7%) of teenagers have an adequate weight. However only 65% of participants perceive themselves normal-weighted; the tendency is to perceive oneself as overweight, in disagreement with the paediatricians’ opinion (overweight self-perception 27,7% vs 11.5% paediatricians). According to paediatricians, male and female opinions are equally reported in the weight category. However, according to personal perception, participants who self-reported overweight are two-thirds females, with a significant difference (Chi square 622,73, df = 2, *p* < .000). Concerning dietary habits, 22,6% of sample declared never to have breakfast at home, and 16,4% declared never to have a snack at school. Moreover, 46,6% of the sample (with a higher proportion of females) do not participate in sports.

2. Internet exposure and sexual behaviour

Adolescents of our sample received their first smartphone at the age of 11 (females earlier than males, 11,3 vs 11,6 f = 36,76; *p* < .001). The use of the internet suggests in many cases a form of dependency. More than half of the sample remains online for longer than they intended. Females more than males appear to be at risk of overuse and dysfunctional use (they claim to remain online longer than desired) (Chi-square 104,02; df = 2; *p* < .001). The use of internet and social networks negatively affects school performance in 70,7% of cases. Although 31,9% of the teenagers declare that the time spent online occasionally influences their studies, for 17,1%, studies are usually negatively conditioned. For males this negative influence is greater than for females (Chi-square 23,42; df = 2; *p* < .001). About half of the sample (48,1%) consider themselves usually able to find reliable information online. Males, more than females, consider themselves always able to find reliable information via Internet (Chi-square 133,40; df = 2; p < .001).

Concerning sexual behaviour, 57,6% of sample declare to have had sexual intercourse with a higher prevalence of males (56,7%) than females. Concerning age, 50% of adolescents aged 13 (*n* = 72 out of 141), with no gender differences, claimed to have had sexual intercourse. The percentage of teenagers with sexual experience grows with age (62,7% at 17 years, 79,3% at 19 years). Among teenagers with sexual experience, 34,4% declare that they never use contraceptives during sexual intercourse. However, the percentage of adolescents who “never” use contraceptives decreases with age, going from 69,4% at 13 years to 46,9% at 15 years to 20% to 19 years of age. Concerning gender, males declare that they use contraceptives more often than females (Chi-square 25,286; df = 2; *p* < .001). 61,4% of the sample declares not to have received any sexual education in the family.

14,4% of the total sample declared to have received sexual proposals from adults by “dating” apps, and 3,2% declared to have offered sex in exchange for payment. Participants who reported to offer sex in exchange for payment are two-thirds males. This difference between males and females is statistically significant (Chi-square 52,59; df = 1; *p* < .001). Table 2 displays the gender distribution of internet exposure and sexual behavior in the study population.

3. Externalizing and internalizing problems

**Table 2:** Frequency and percentage for internet exposure and sexual behavior variables according to gender

		Males n %	Females n %	Total n %
Have you ever remain online longer than you expected?	never	594	386	980
		60,60%	39,40%	*8,50%*
	occasionally	2371	2125	4496
		52,70%	47,30%	*39%*
	regularly	2759	3293	6051
		45,60%	54,40%	*52,50%*
Is your study negatively influenced by on-line activities?	never	1648	1727	3375
		48,80%	51,20%	*29,30%*
	occasionally	2996	3178	6174
		48,50%	51,50%	*53,60%*
	regularly	1080	898	1978
		54,60%	45,40%	*17,20%*
Do you believe yourself able to find online reliable information?	never	483	568	1051
		46,00%	54,00%	*9,10%*
	occasionally	2175	2748	4923
		44,20%	55,80%	*42,70%*
	regularly	3066	2487	5553
		55,20%	44,80%	*48,20%*
Have you ever had sexual intercourse?	No	1963	2925	4888
		40,20%	59,80%	*42,40%*
	yes	3761	2878	6639
		56,70%	43,30%	*57,60%*
Do you use contraceptive methods?	never	1205	1082	2287
		52,70%	47,30%	*34,40%*
	occasionally	717	461	1178
		60,90%	39,10%	*17,70%*
	regularly	1839	1335	3174
		57,90%	42,10%	*47,80%*
Did you have a sexual education in family?	No	3531	3552	7083
		49,90%	50,10%	*61,40%*
	yes	2193	2251	4444
		49,30%	50,70%	*38,60%*
Have you ever received sexual proposed by adults via the meetings app.?	never	4943	4929	9872
		50,10%	49,90%	*85,60%*
	occasionally	551	670	1221
		45,10%	54,90%	*10,60%*
	regularly	230	204	434
		53,00%	47,00%	*3,80%*
Have you ever give sexual service in exchange of payment?	never	5474	5687	11,161
		49,00%	51,00%	*96,80%*
	occasionally	250	116	366
		68,30%	31,70%	*3,20%*
	regularly	0	0	0
		0,00%	0,00%	*0%*

Note: Percentage are reported for males and females while percentage in *italic* are reported for total sample

36,8% of the sample declares that they smoke cigarettes and in 17% of cases they smoke regularly. The percentage of female smokers is significantly higher than males (Chi-square 31,13; df = 2; *p* < .001).

Concerning binge drinking, although 61.3% of the participants report never binge drinking, 8,8% declare regular binge drinking. Males reported binge drinking behaviour more frequently than females (55,9% vs. 44,1%) (Chi-square 22,51; df = 2; *p* < .001).

Regarding drug use, 22.3% of the sample claims they have used drugs at least once and in 6,5%, the consumption has been regular. The intake of drugs is more frequent in males (Chi-square 47,36; df = 2; p < .001).

6687 participants affirmed to have been victims of bullying or cyberbullying. 33,7% of the sample reported to have been bullied, and 12,6% reported having been cyberbullied at least once. In 67% of cases, bullied or cyberbullied teenagers did not talk with anyone about their victimization.

The percentage of females who reported to “regularly” be victims of bullying and cyberbullying episodes is significantly higher than males (victim of bullying Chi-square 37,09; df = 2; *p* < .001; victim of cyberbullying Chi-square 110,59; df = 2; *p* < .001). The percentage of males who reported to “regularly” be bullied or cyberbullied is significantly higher than females (Chi-square 23,24; df = 2; *p* < .001).

15,3% of the sample (*n* = 1759 teenagers) claims to have intentionally self-injured at least once. Females reported self-harm significantly more often than males, occasionally and regularly (Chi-square 104,03; df = 2; p < .001). 6% of the sample reported to “regularly” feel intense psychological suffering. Females reported this regular suffering three times more than males did (chi square 331; df = 2; p < .001). Table 3 displays the geneder distribution of internalizing and externalizing problems.

4. Family context

**Table 3:** Frequency and percentage for externalizing and internalizing problems variables according to gender

		Males n %	Females n %	Total n %
Do you smoke cigarettes?	never	3792	3565	7357
		51,50%	48,50%	*63,80%*
	occasionally	999	1209	2208
		45,20%	54,80%	*19,20%*
	regularly	933	1029	1962
		47,60%	52,40%	17%
Have you ever been a binge drinker?	never	3414	3656	7070
		48,30%	51,70%	*61,30%*
	occasionally	1744	1701	3445
		50,60%	49,40%	*29,90%*
	regularly	566	446	1012
		55,90%	44,10%	*8,80%*
Have you ever been a drug user?	never	4306	4650	8956
		48,10%	51,90%	*77,70%*
	occasionally	977	851	1828
		53,40%	46,60%	*15,90%*
	regularly	441	302	743
		59,40%	40,60%	*6,40%*
Have you ever been a victim of bullism?	never	3936	3705	7641
		51,50%	48,50%	*66,30%*
	occasionally	1519	1721	3240
		46,90%	53,10%	*28,10%*
	regularly	269	377	646
		41,60%	58,40%	*5,60%*
Have you ever been a victim of cyberbullism?	never	5188	4884	10,072
		51,50%	48,50%	*87,40%*
	occasionally	426	752	1178
		36,20%	63,80%	*10,20%*
	regularly	110	167	277
		39,70%	60,30%	*2,40%*
Have you ever been a bully or cyberbully?	never	3704	3986	7690
		48,20%	51,80%	*66,70%*
	occasionally	1731	1590	3321
		52,10%	47,90%	*28,80%*
	regularly	289	227	516
		56,00%	44,00%	*4,50%*
Have you ever feel an intense psychological sufferance?	Never	1703	777	2480
		68,7%	31,3%	*67,9%*
	Occasionally	398	556	954
		41,7%	58,3%	*26,12%*
	regularly	57	161	218
		26,1%	73,9%	*6%*
Have you ever experimented self-harm?	never	5045	4723	9768
		51,60%	48,40%	84,70%
	occasionally	521	791	1312
		39,70%	60,30%	*11,40%*
	regularly	158	289	447
		35,30%	64,70%	*3,90%*

Note: Percentage are reported for males and females while percentage in italic are reported for total sample

Concerning the residential situation, about 8 adolescents out of 10 live with both parents, while 1 in 10 live in single-parent families. There are also 3,8% of the sample living in reconstituted families (own parent and his/her new partner) and a 3,2% living in an “other” situation. 3,4% of the sample declare to have a family made up of same-gender parents. 6,6% of the sample have lived for a short period in a shelter house.

22% of participants declared to regularly be witness to their parents’ quarrels. A quarter of the participants reported at least one physical and/or psychological aggression episode in the family context. In most cases (61,7%), this aggression was carried out by the parents (the father 3 times more frequently than the mother), but the percentage of teenagers who report aggression by their brothers (11,4%), uncles (3,6%) or grandparents (3,6%) is significant. 13,4% of young people report that they have been victims of physical violence in their families, which in 2,9% of cases happens regularly.

Gender differences emerged in the family climate with females reporting to be witnesses of parental quarrels more often than males (Chi square 179,89 df.2; *p* = .001), reporting a higher degree of family aggression (Chi square 65,81; df.2; *p* < .001), and stating to be victims of physical and psychological aggression more than males (Chi square 19,45; df.2; p = .001).

The relationship with parents is defined globally positive, with the mother receiving a more positive evaluation. The relationship with the father is negatively evaluated in 18% of the sample, while that with the mother in only 8,9%. Regarding the sensitive issues that worry teenagers and communication in the family, 46,8% declare that they usually turn to their parents to talk about things that worry them. Females declare that they turn more to their parents in case of difficulty than males (Chi square 18,30; df. 2; *p* < .001). Table 4 displays the gender distribution of family context variables.

5. Social context

**Table 4:** Frequency and percentage for family context variables according to gender

		Males n %	Females n %	Total n %
Who do you live with?	Both parents	4802	4802	9604
		50,0%	50,0%	*83,3%*
	Mother	438	526	964
		45,4%	54,6%	*8,4%*
	Father	79	67	146
		54,1%	45,9%	*1,3%*
	1 parent + a new partner	210	233	443
		47,4%	52,6%	*3,8%*
	Other	195	175	370
		52,7%	47,3%	*3,2%*
Your family consists of:	Same gender parents	183	214	397
		46,1%	53,9%	*3,4%*
	Different gender parents	5223	5208	10,431
		50,1%	49,9%	*90,5%*
	Single parent	237	300	537
		44,1%	55,9%	*4,7%*
	Other	81	81	162
		50%	50%	*1,4%*
Have you ever lived in a shelter House?	No	5318	5452	10,770
		49,40%	50,60%	*93,40%*
	Yes	406	351	757
		53,60%	46,40%	*6,60%*
Have you ever been a testimonial of argue in family?	never	1679	1309	2988
		56,20%	43,80%	*25,90%*
	occasionally	3067	2938	6005
		51,10%	48,90%	*52,10%*
	regularly	978	1556	2534
		38,60%	61,40%	*22%*
Have you ever been a testimonial of Violence in family?	never	4429	4221	8650
		51,20%	48,80%	*75,00%*
	occasionally	996	1063	2059
		48,40%	51,60%	*17,90%*
	regularly	299	519	818
		36,60%	63,40%	*7,10%*
Have you ever been a victim of Violence?	never	4933	5050	9983
		49,40%	50,60%	*86,60%*
	occasionally	652	554	1206
		54,10%	45,90%	*10,50%*
	regularly	139	199	338
		41,10%	58,90%	*2,90%*
How do you describe your relationship with your father?	negative	451	591	1042
		43,30%	56,70%	*18,00%*
	neutral	430	430	860
		50,00%	50,00%	*14,80%*
	positive	2157	1735	3892
		55,40%	44,60%	*67,20%*
How do you describe your relationship with your mother?	negative	276	248	524
		52,70%	47,30%	*8,90%*
	neutral	296	359	655
		45,20%	54,80%	*11,20%*
	positive	2494	2192	4686
		53,20%	46,80%	*79,90%*
Do you turn to your parents in case of difficulty?	never	819	748	1567
		52,30%	47,70%	*13,60%*
	occasionally	2343	2230	4573
		51,20%	48,40%	*39,70%*
	regularly	2562	2825	5387
		47,60%	52,40%	*46,70%*

Note. Percentage are reported for males and females while percentage in *italic* are reported for total sample

Table 5 shows the gender distribution of social context variables in the study population. The social context, according to teenagers, allows them to have complete freedom of expression. This freedom is “usually” perceived in 51,7% of cases. Moreover, males, more than females, believe that social context allows freedom of expression (Chi square 78,88; df. 2; *p* < .001). The school seems distant from the teenagers’ need; about half of the sample (51,4%), in fact, reports that the school is “never” receptive to the problems of teenagers. Males, more than females, report positive opinions on the attention of the school to the problems of boys (Chi square 18,30; df. 2; *p* < .001). The teenagers of the sample claim to have needed psychological support in 50,6% of cases, but only in 15,8% of cases this need was matched by a real psychological evaluation. Females, compared to males, reported a greater need for psychological support (Chi square 467,03; df 2; *p* < .001), as well as a greater use of psychological services (Chi-square 75,61; df 1; p < .001). 

More support seems to come from friends. Most of the teenagers (58,3%) declared that they usually turn to friends in case of problems and receive help from them in case of difficulty (70,2%). Females reported that they talk with friends in case of problems more “regularly” than males (Chi square 170,03; df = 2; *p* < .001), and that they receive help from them (Chi-square 33,83; df = 2; p < .001). In moments of loneliness, 35,8% of teenagers report using internet or social networks to talk to others, and in females this happens more frequently (Chi square 245,30; df = 2 p < .001).

Social relationships take place both in informal and formal groups. 35,7% of the sample (*n* = 4113), in fact, claims to be members of youth associations. The most frequently attended associations are scouting (secular or religious) (37,7%), sport (18,2%), or regional traditions (10,1%).

Real social relationships are usually preferred to virtual ones, but about 1 adolescent in 10 (9,2%) declares to “always” prefer online interactions rather than face to face. There is no difference between males and females.

**Table 5:** Frequency and percentage for social context variables according to gender

		Males n %	Females n %	Total n %
Do you believe that the social context allow you freedom of expression?	never	434	381	815
		53,3%	46,7%	*7,1%*
	occasionally	2127	2629	4756
		44,7%	55,3%	*41,3%*
	regularly	3163	2793	5956
		53,1%	46,9%	*51,7%*
Do you believe that the school is receptive to the youth needs?	never	1124	1062	2186
		51,4%	48,6%	*19,0%*
	occasionally	3365	3632	6997
		48,1%	51,9%	*60,7%*
	regularly	1235	1109	2344
		52,7%	47,3%	*20,3%*
Have you ever felt the need for psychological support?	Yes	3371	2323	5694
		59,2%	40,8%	*49,4%*
	No	1768	2286	4054
		43,6%	56,4%	*35,2%*
	In past	585	1194	1779
		32,9%	67,1%	*15,4%*
Have you ever consult a psychologist?	No	4352 52,1%	4007 47,9%	8359 84,2%
	Yes	631 40,1%	942 59,9%	1573 15,8%
Do you turn to your friend in case of problems or difficulties?	never	679	393	1072
		63,3%	36,7%	*9,3%*
	occasionally	2024	1712	3736
		54,2%	45,8%	*32,4%*
	regularly	3021	3698	6719
		45,0%	55,0%	*58,3%*
Do you receive help from your friends in case of need?	never	245	223	468
		52,4%	47,6%	*4,1%*
	occasionally	1598	1360	2958
		54,0%	46,0%	*25,7%*
	regularly	3881	4220	8101
		47,9%	52,1%	*70,3%*
Do you use internet to talk with other people?	never	1467	923	2390
		61,4%	38,6%	*20,7%*
	occasionally	2544	2461	5005
		50,8%	49,2%	*43,4%*
	regularly	1713	2419	4132
		41,5%	58,5%	*35,8%*
Are you a member of a youth associations?	No	3933	3993	7926
		49,6%	50,4%	*68,8%*
	Yes	1791	1810	3601
		49,7%	50,3%	*31,2%*
If yes, which association are you a member of?	scout	217	218	435
		49,9%	50,1%	*10,6%*
	Religious association	486	630	1116
		43,5%	56,5%	*27,1%*
	Sport associations	247	502	749
		33,0%	67,0%	*18,2%*
	Traditional association	237	179	416
		57,0%	43,0%	*10,1%*
	Other	920	477	1397
		65,9%	34,1%	*34,0%*

Note. Percentage are reported for males and females rows while percentage in *italic* are reported for total sample

## Discussion

The survey proposed in this paper provides new and interesting data about the characteristics of Italian teenagers, underlining the importance of prevention, support, information and training of all professionals who deal with physical and mental health of childhood.

Concerning body perception, although according to paediatricians 8 teenagers out of 10 have an adequate weight, only 6 out of 10 perceive themselves as normal-weight. Teenagers tend to perceive themselves as overweight. This tendency is higher in females compared to males. Moreover, females report dietary restrictions aimed at reducing intake and, consequently, reducing their weight. Young age, female gender, overweight misperception, and being on a diet are some of the few identified risk factors that have been reliably linked to the development of eating disorders [17, 18]_._ Thus, some teenagers may progress from initial attempts to control their weight to unhealthy dietary restrictions, even to starvation. Weight loss is often reinforced by media models of shape, and peer and family environments, but an excessive preoccupation with body imagine can lead to incomplete eating disorders and other psychopathological problems (social isolation, irritability, difficulty of concentrating). Body dissatisfaction and desire of a thinner figure are described in young boys and girls, and early teenagers may endorse an image of a thin body model, promoted by media, as a desirable standard to achieve [19].

The findings of this research are consistent with literature, pointing out the importance of carrying out preventive interventions on eating disorders, in terms of dissemination of indications for a correct diet in adolescence, and in interception of populations at risk.

The second part of the study was aimed at investigating internet exposure and sexual behaviours. Our results showed that Italian adolescents received their first smartphones at the age of 11. As digital natives, adolescents use smartphones to express their thoughts in an online space and search for emotional relationships in the pursuit of instant reactions and feedback. However, given their immature competence of control, teenagers resulted to be at high risk of smartphone addiction. Paediatricians should encourage parents to think about giving their children a smartphone at an early age, given that this instrument seems to be difficult to handle. Teenagers (especially females) seem to be at risk of overuse and dysfunctional use of smartphones (they claim to regularly remain online longer than desired) and this negatively affects school performance.

With respect to sexual habits, our findings showed a certain precociousness of activation in this area which is accompanied by reduced awareness of risks, and consequent poor use of contraceptives. The sample shows how, even today, sex education in our country does not occur in the family or school contexts, but rather according to the indications of peers, which do not ensure adequate education. These data invite us to reflect on how important it is for paediatricians and all people who work with youth to promote interventions to help the parents of pre-adolescent and adolescent children give correct sex education to their children [29, 30]_._

An alarming fact is that 3,2% of teenagers have had sexual experiences to gain economic benefits and 14,4% have received sexual proposals by adults, mainly through social networks or dating apps. Once more, these data suggest the risk of internet exposure for Italian youth. In fact, although the adolescents consider themselves able to find reliable information online, our findings suggest that they are not able, given that they received sexual proposals by adults via apps, and they reported to be victims of cyberbullying.

The third part of the study was aimed at increasing our knowledge of externalizing and internalizing problems. Concerning drug consumption, our study showed that 2 adolescents out of 10 had consumed drugs at least once. Moreover, 6,5% of teenagers stated a regular consumption of drugs, and 8,8% declared regular binge drinking. Both the regular binge drinking and drug consumption are more frequent in males than females.

The relevance of these data is given by the consequences that repeated binge drinking and drug consumption have on an adolescent’s health. Early and repeated binge drinking, in fact, is associated to a decay of the attentive function and a decrease of cognitive performance in verbal learning and short-delay memory tasks with a linear dose-dependent relationship. Further, substance exposure during adolescence may adversely affect cognitive functioning, and marijuana users have attention deficit [20], poorer performance on working memory, verbal learning and memory. Moreover, poorer performance in visuospatial functioning and psychometric motor speed tasks have been observed with higher doses and an earlier age of onset [21–23]. In literature, the use of cannabis has been confirmed as a risk factor to use other illicit drugs [24]. Our data also show that females report a higher frequency of cigarette smoking than males. These data are particularly interesting given the close link between the precocity of this behaviour and the increased risk of tobacco dependence with consequent long-term effects [25]. Even a brief exposure to nicotine during the adolescent period can have long-lasting effects on neurochemistry and behaviour, including decreased cognitive function, and could enhance drug reward [26, 27]_._ Early intervention should be developed, especially in the female population, which, according to our data, resulted to be at higher risk. In fact, epidemiological data have consistently demonstrated that women have less success quitting tobacco use, a higher risk of developing tobacco-related disease, and are especially sensitive to the adverse effects of nicotine withdrawal [28]_._

Concerning bullying, three adolescents out of ten reported to have been bullied, and one of ten reported being cyberbullied at least once in their lives. In most of these cases, bullied or cyberbullied teenagers did not talk with anyone about their victimization. Females are more frequently victims of bullying and cyberbullying, while males tend to assume the bully or cyberbully role. Literature suggests that bullying experiences are typically confined to school, and that the negative consequences of involvement in face-to-face bullying involves psychosocial adjustment [30] and perceptions of school [31]. Moreover, victims of cyberbullying report that they are afraid to go to school [32] and, for some young people, this fear escalates into active avoidance manifested as truancy [33]. Involvement in cyberbullying also results in young people feeling less safe in school [34] and having negative attitudes towards school [35]. Thus, paediatricians should pay particular attention to symptoms of school avoidance and fear of school reported by parents or teenagers, given that they could be signs of bullying.

Finally, 15% of adolescents claimed to have intentionally self-injured at least once, and females reported this behaviour more frequently than males, accompanied by psychological suffering. Referred to in literature and media as “self-injurious behaviour,” “self-injury,” “self-harm,” “self-mutilation,” or “cutting”, self-injury is typically defined as the deliberate, self-inflicted destruction of body tissue without suicidal intent and for purposes not socially sanctioned [36]. Research has shown that the location of the self-injury is usually in areas that are hidden by clothing, such as the arms, abdomen, inner thighs, feet, genitals, and torso (especially near the breasts in females). Our results confirm previous results about the reason for self-injuring in terms of reducing psychological pain and expressing and alleviating psychological distress [37]. Given that it has been noted that self-harm is statistically associated with an increased risk of suicide and can result in unanticipated severe harm or fatality [38], paediatricians should be pay attention to cuts or signs of self-harm during medical visits, when teenagers remove their clothes. Future research should explore the family context of adolescents who report self-harm.

Concerning the family context, it must be noted that one in five teenagers are witness to physical or psychological aggression in the family of origin. A lot of research has provided evidence that the parenting context has important implications for teenagers’ involvement. Adolescent males who are victims or witnesses of violence exhibit a higher rate of violence perpetration, and a lower level of self-control [39]. On the contrary, adolescent females who are victims are significantly more likely to attempt suicide and self-harm than males. It is estimated that five to six times as many adolescent girls self-harm as compared to boys [40]. Moreover, exposure to violence increases vulnerability to a broad range of mental and physical health problems over the life course; exposure to physical abuse in childhood is associated with a 54% increase in odds of depressive disorder, a 78% increase in odds of risky sexual behaviour, and a 32% increase in odds of obesity. However, despite the relevance of this phenomenon for mental and physical health in adolescents, only a small percentage of these violent incidents are reported to law enforcement, health care clinicians, or child protection agencies. The most effective violence prevention strategies include parent and family-focused programs, early childhood education, school-based programs, therapeutic or counselling interventions, and public policy [41]. Thus, paediatricians should pay particular attention to symptoms of family conflict and violence and, when they think that there is a risk for a negative family environment, they should explore this theme in a private talk with the teenager [42].

Our data demonstrated that, in the adolescent’s perception, the school seems distant and not receptive to the teenager’s needs and problems. This school inadequacy is suffered in a greater measure by girls.

Further, adolescent girls today suffer from internalizing problems, such as somatic symptoms and mental health problems, at higher rates compared to those of previous decades [1, 43].

Research shows that mental health problems are currently a public health challenge globally [42] and affect 10–20% of children and teenagers worldwide [2]. Therefore, we encourage schools to promote an enhanced mental health literacy.

Paediatricians and schools should be equipped with the elements to encourage young people to grow up in an environment where they can feel free to express themselves, and to be listened to, without fear of being judged [44].

In our sample, more than half the teenagers felt the need for psychological support, but only in 15% of cases was this need met. It is critical that teachers and health professionals be trained to recognize signs and symptoms of psychological distress and children at risk.

The present study should be considered in the light of some limitations. First, this survey analysed only the gender difference neglecting the role of other demographic variables (such as age) may have in promoting different lifestyle. Further research should deep this aspect analysing how the internalizing and externalizing problems vary according to age. Second, this study did not consider the relationship between adolescent lifestyle and family or peer context. Thus, it is not possible, for instance, to know how the family context affects internalizing and externalizing problems in Italian adolescents. In the same direction, our findings did not allow us to understand how the social context (school, peer group both formal or informal) influences the internalizing and externalizing behaviours. Future research should explore the interrelation among variables. However, despite these limitations, this study is very interesting, given that it offers insight into internalizing and externalizing problems in teenagers involving a large population sample from the whole Italian territory.

## Conclusions

This study consisted in a survey of Italian adolescents and, to our knowledge, it is the first investigation that enrolled more than 11,000 teenagers living in the northern, central, southern, and island areas of Italy. Moreover, the findings of this study provide useful indications for health professionals and school authorities to promote health programs. First, a misperception of body image and an overweight perception, especially in female adolescents, constituting a risk factor for subsequent eating disorders, should be attentively evaluated and discussed with youth in clinical and school settings. Second, the consequences of adolescents’ smartphone use should be discussed with parents in clinical and school settings. In fact, precocious access to a personal smartphone and the youth’s immature control competence increase the risk of dependency more than smartphone-related health risk behaviours concerning sexual activities and cyberbullying.

Moreover, the presence of health risk behaviours, such as binge drinking and drug consumption, should encourage the promotion of specific intervention programs. The presence of inner suffering and the need for psychological support that adolescents reported was not communicated to school authorities or health professionals, and merged into self-harm behaviours that constitute a significant risk factor for subsequent suicide attempts. Thus, improving psychological services inside the school context would constitute a protective factor to prevent suffering. The early detection of self-harm behaviour would allow early intervention.
